# Evaluation of Subfoveal Choroidal Thickness in Internal Carotid Artery Stenosis

**DOI:** 10.1155/2016/5296048

**Published:** 2016-02-18

**Authors:** Betül İlkay Sezgin Akçay, Esra Kardeş, Sultan Maçin, Cihan Ünlü, Engin Bilge Özgürhan, Aydın Maçin, Tahir Kansu Bozkurt, Ahmet Ergin, Reyhan Surmeli

**Affiliations:** ^1^Ophthalmology Clinic, Ümraniye Research and Training Hospital, Istanbul, Turkey; ^2^Radiology Clinic, Ümraniye Research and Training Hospital, Istanbul, Turkey; ^3^Beyoğlu Research and Training Hospital, Ophthalmology Clinic, Beyoğlu Research and Training Hospital, Istanbul, Turkey; ^4^Neurology Clinic, Ümraniye Research and Training Hospital, Istanbul, Turkey

## Abstract

*Purpose*. To evaluate the relationship between internal carotid artery (ICA) stenosis and subfoveal choroidal thickness (SFCT) in the elderly population.* Methods*. A total of 42 eyes of 21 patients with more than 70% ICA stenosis (Group 1) on one side and less than 70% stenosis (Group 2) on the other side were recruited for this study. ICA stenosis was diagnosed using both the B-mode and Doppler ultrasound. The two groups were compared in terms of the percentage of stenosis, SFCT measurements, intraocular pressure, ocular perfusion pressure, refractive error, and peak systolic velocity. Eyes were examined with the RTVue-100 OCT device by the EDI-OCT technique.* Results*. The mean age of the patients was 71.9 ± 10.8 years. The mean percentage of ICA stenosis was 74 ± 4.9% in Group 1 and 47.5 ± 7.7% in Group 2. The mean SFCT was 231.9 ± 44.6 *μ*m in Group 1 and 216.2 ± 46.8 *μ*m in Group 2, which was significantly lower (*P* = 0.028). A statistically significant positive correlation was found between the percentage of internal carotid artery stenosis and SFCT (*r* = 0896, *P* = 0.001).* Conclusions*. Compensatory SFCT increase can be seen in ipsilateral internal carotid artery stenosis greater than 70%.

## 1. Introduction

The choroid is a highly vascularized structure. Ninety-five percent of the blood flow into the eye supplies the uveal structures, and the choroid receives more than 70% of the portion that enters the uveal structures [1]. The ophthalmic artery, the first branch of the internal carotid artery (ICA), divides to form the central retinal artery and the posterior ciliary artery, which are responsible for the nourishment of the posterior choroid [2, 3]. The choroid lies between the lamina fusca of the sclera and the retinal pigment epithelium [3]. The main function of the choroid is to provide oxygen and nourishment to the outer retinal layers and uveal structures [4].

The* in vivo* structure of the choroid cannot be visualized properly with conventional methods such as fundus photography and fluorescein angiography due to the pigments in the retinal pigment epithelium (RPE) that attenuate the incident light. Indocyanine green angiography allows better imaging of the choroidal vessels, but it does not provide cross-sectional data. Recently, Spaide et al. demonstrated that choroidal thickness could be measured successfully and noninvasively* in vivo* using commercially available spectral-domain optical coherence tomography (SD-OCT) devices that use the enhanced depth imaging (EDI) technique [5].

Severe stenosis of the extracranial segment of internal carotid artery (ICA) is the main reason for visual disturbances associated with ocular ischemia [6, 7]. Acute transient monocular blindness due to entrapment of emboli in the retinal arterial system is the most common ischemic ocular symptom (30–40%) [8]. Reported range of chronic progressive ocular ischemia in patients with carotid artery stenosis or occlusion is 5–21% [9–12]. Extracranial carotid artery stenosis of more than 50% is present in 1.5 to 9% of the population, with a higher incidence in the elderly, and 70% stenosis of the ICA is the threshold currently used for surgical intervention [13, 14]. An embolus originating in the carotid artery is likely to enter the ophthalmic artery, which is the first branch of the artery, leading to hypoperfusion of the retina and choroid. Choroidal thickness has attracted a great deal of interest in recent years to find out its association with many variables including age, sex, smoking, and disease states in recent studies [15–23]. However, extensive literature research did not reveal any information about effect of internal carotid artery stenosis on subfoveal choroidal thickness. Ocular ischemic syndrome is one of the most severe clinical entities associated with the carotid artery stenosis. Therefore, we aimed to evaluate whether the ICA stenosis of more than 70% has an effect on the ipsilateral subfoveal choroidal thickness before the onset of a symptomatic ischemic process by comparing to the opposite eye.

## 2. Methods

In this study, twenty-one patients with a diagnosis of ICA stenosis greater than 70% on only one side (the opposite ICAs had less than 70% stenosis) were included at the Radiology Clinic of the Ümraniye Research and Training Hospital between November 2013 and November 2014. The eyes were divided into two groups with respect to the degree of stenosis. The eyes ipsilateral to the ICA with greater stenosis (>70%) were recruited as Group 1, and the opposite eyes (which were ipsilateral to the ICA with less than 70% stenosis) were included in Group 2. The two groups were compared in terms of the percentage of ICA stenosis, peak systolic velocity of ICA, SFCT measurements, intraocular pressure, and ocular perfusion pressure. In addition, the correlation between the percentage of ICA stenosis and the thickness of the subfoveal choroidal tissue was evaluated. The present study was performed according to the principles of the Declaration of Helsinki. Informed consent was obtained from each patient after a detailed explanation of the study. Patients were excluded if they met any of the following criteria: a history of previous retinal disease, media opacity that prevented OCT imaging, intraocular surgery within six months, glaucoma, high myopia with a refractive error greater than 6 diopters, or other eye diseases that could compromise the visual acuity. In addition, to avoid confounding by other known factors that can affect choroidal thickness, patients with uncontrolled hypertension (HT), as defined by a blood pressure > 140/90 mmHg or the use of antihypertensive medication, and diabetes mellitus (DM) were excluded.

All patients underwent a baseline examination including refraction, best-corrected Snellen visual acuity, a dilated fundus and slit-lamp examination, and enhanced depth imaging optical coherence tomography (EDI-OCT). Detailed medical histories were obtained including HT, hypercholesterolemia, and smoking and history of cardiovascular or cerebrovascular disease. Smoking history was evaluated in pack years (1 pack year = 20 cigarettes/day for one year).

Systolic and diastolic blood pressure were measured using an automated sphygmomanometer (BP-203 RVIIIB; Omron Healthcare Co., Ltd., Kyoto, Japan), based on the cuff oscillometric method, with an appropriately sized cuff on the right arm in the sitting position after resting for ≥5 minutes before the OCT measurement. The mean of 3 measurements was used for the analysis. The mean arterial blood pressure (MABP) was calculated as mean arterial blood pressure = 2/3 diastolic blood pressure +1/3 systolic blood pressure. The mean ocular perfusion pressure was calculated as mean ocular perfusion pressure = 2/3 mean arterial blood pressure – IOP.

### 2.1. Gray-Scale and Doppler US Examination

ICA stenosis was diagnosed using both the B-mode and Doppler ultrasound (Aplio MX, Toshiba Medical Systems, Corp., Otawara, Japan) methods with a 7.5 MHz linear ultrasound probe by an experienced radiologist (SM). Colored Doppler US was performed both on and distal to the plaque, which was diagnosed by B-mode ultrasound with gray scale in the longitudinal plane. Spectral Doppler flow was measured within the areas that showed jet flow, heterogeneous color patterns, and abnormal flow of narrowed areas. A consensus of the panel was used to develop the criteria for the table of recommended Doppler thresholds for the diagnosis of ICA stenosis [24]. Both the degree of stenosis in the ICA and the Peak Systolic Velocity (PSV) were recorded.

### 2.2. OCT Protocol and Choroidal Thickness Measurement

**Figure 1:**
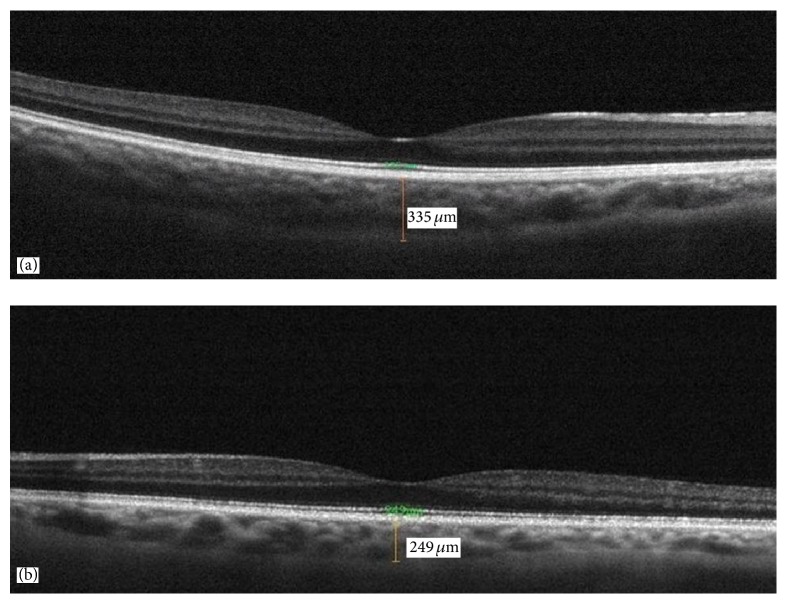
Sixty-four-year-old male patient: (a) 335 *μ*m subfoveal choroidal thickness in right eye (85% stenosis in right internal carotid artery) and (b) 249 *μ*m subfoveal choroidal thickness in left eye (55% stenosis in left internal carotid artery).

All eyes were examined with the RTVue-100 OCT device (Optovue Inc., Fremont, CA) by the EDI-OCT technique. The RTVue-100 OCT device utilizes a choroid mode in which the OCT image is automatically inverted so that the chorioretinal interface is adjacent to zero delay. The line protocol of OCT was performed in this mode. The line protocol performed in the choroid mode included a horizontal single line image through the foveal center and was derived from an average of 16 frames (each frame consisted of 1024 A-scans, and a total of 16,384 data points were averaged to a single scan image). The measurements were done in 1 : 1 microns mode. The subfoveal choroidal thickness measurements were performed manually using the caliper function of the OCT device. The SFCT was defined as a vertical line perpendicular to the center of the fovea from the outer portion of the hyperreflective line of Bruch's membrane to the choroidoscleral interface (Figures 1(a) and 1(b)). All OCT examinations were performed by the same experienced technician, and the SFCT measurements in the current study were performed by a retina specialist who was blinded to the group designations (CU).

### 2.3. Statistical Analysis

All data were analyzed using SPSS Version 18.0 (SPSS, Inc., Chicago, IL). Data are expressed as the means ± standard deviation of the mean. The Kolmogorov-Smirnov test was used to identify the normality of distribution. Student's *t*-test was used to compare parameters between groups. Spearman rank correlation coefficient was used to evaluate associations between the measured variables. Degrees of agreement between examiners was assessed by calculating intraclass correlation coefficients (ICC) and 95% confidence intervals (CI). Results with a *P* value <0.05 were considered to be significant.

## 3. Results

Forty-two eyes of 21 patients (13 males and 8 females) with more than 70% carotid artery stenosis on one side and less than 70% stenosis on the other side were recruited for the study. The mean age of the patients was 71.9 ± 10.8 years (with a range of 52–84 years). The mean ICA stenosis was 60.7 ± 14.6 *μ*m for all of the patients. The characteristics and demographic features of the patients are shown in Table 1.

There was a statistically significant difference in the mean SFCT value between Group 1 (231.9 ± 44.6 *μ*m) and Group 2 (216.2 ± 46.8 *μ*m) (*P* = 0.028). However, the refractive error and mean IOP and OPP values were not significantly different between the two groups (*P* = 0.530, *P* = 0.546, and *P* = 0.430, resp.).  Table 2 shows the subfoveal choroidal thickness and other clinical measurements of the two groups.

**Figure 2:**
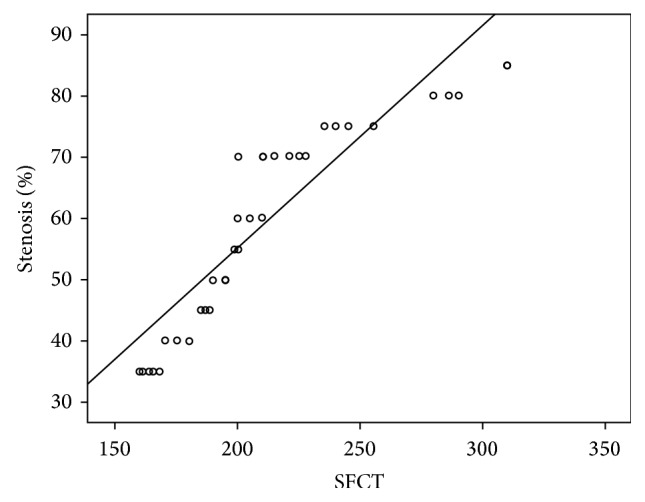
Scatter plot graph showing the relationship percentage of internal carotid artery stenosis and subfoveal choroidal thickness (SFCT).

There was a statistically significant positive correlation between the percentage of internal carotid artery stenosis and SFCT (*r* = 0.896, *P* = 0.001). This was shown in scatter plot with regression line (Figure 2). However, the SFCT value was not significantly associated with the IOP or OPP (*r* = 0.089, *P* = 0.693 and *r* = 0.009, *P* = 0.726, resp.).

A correlation analysis between the SFCT and the percentage of ICA stenosis, IOP, and OPP of the whole cohort is shown in Table 3.

The mean SFCT measurements in the current study were performed by two independent examiners BS [Examiner 1] and CU [Examiner 2]. The mean subfoveal choroidal thicknesses measured by examiner 1 and examiner 2 were 223.5 ± 46.8 *μ*m and 226.2 ± 47.1 *μ*m, respectively. The level of agreement between examiner 1 and examiner 2 was 96% (ICC: 0.969; 95% CI: 0.956–0.982; *P* < 0.01).

## 4. Discussion

The ophthalmic artery, which is the first branch of the internal carotid artery, branches to form the central retinal artery and the posterior ciliary arteries. In 90% of eyes, the posterior choroid receives its blood supply from two major posterior ciliary arteries: the medial and lateral posterior ciliary arteries. The long posterior ciliary arteries supply blood to the anterior uvea. Smaller short posterior ciliary arteries pass to the posterior part of the eyeball around the optic nerve and supply choroid and ciliary processes [3].

When the ICA is obstructed, blood flow to the brain is maintained by the opening of collateral channels between the terminal external carotid artery branches and the terminal branches of the ophthalmic artery. The opening up of ECA-ICA collateral channels via the ophthalmic artery can cause many signs and symptoms such as delay/asymmetry of ECA pulses, angular, brow, cheek pulses, frontal artery sign, dilated episcleral arteries, and Olivarius' external carotid sign. Retrograde flow to the ICA occurs through these channels via the ophthalmic artery, resulting in hypoperfusion of the retinal and choroidal circulation [25]. This phenomenon may lead to compromised choroidal flow and result in photoreceptor dysfunction. Structurally and functionally, normal choroidal vasculature is crucial for proper retinal function. Numerous prospective, randomized, and multicenter studies have been designed to evaluate choroidal thickness in normal and disease states [15–23]. However, no clear evidence has been found regarding the effect of internal carotid artery stenosis on choroidal thickness.

The present study was designed to evaluate whether ICA stenosis of more than 70% has an effect on ipsilateral subfoveal choroidal thickness compared to the opposite eye. Our data indicate that the SFCT is significantly thicker in eyes on the side with more than 70% stenosis (231.9 ± 44.6 versus 216.2 ± 46.8, *P* = 0.028).

When stenosis occludes at least 70% of the lumen, or if the residual lumen diameter is ≤ 1.5 mm, a carotid bruit is usually heard at the origin of the ICA [26]. One of the most fundamental principles of the circulation is the ability to control its own blood flow in proportion to its metabolic requirements. Long term control of local blood flow means slow changes over a period of time resulting in change in physical size and number of the blood vessels. One of the most important mechanisms of the long term regulation is the change in the vascularity, as if when the metabolism of a given tissue increases vascularity increases. Angiogenesis is also an important local blood control mechanism in order to protect tissues from ischemia. Inadequate blood supply leads to release of the angiogenic factors which further leads to promoting new vessel formation. Development of collateral formation is also an important long term blood flow regulation [27]. Concerning the high metabolic demand of the choroid, increased SFCT that we observed at the stenosis site might be a dilatation in the choriocapillaris vasculature to prevent retinal and choroidal ischemia as a result of the diminished blood flow due to ICA stenosis.

In accordance with our results, Salazar et al. investigated alterations in the choroid in hypercholesterolemic rabbits and concluded that the choroid was thicker in the presence of hypercholesterolemia [28]. Similarly, Wong et al. reported that the mean SFCT was thicker among patients with hypercholesterolemia [29]. Altinkaynak et al. evaluated the SFCT measured by EDI-OCT in the eyes of chronic heart failure (CHF) patients. In contrast to our results, they found that the mean SFCT was 181.2 ± 80.23 *μ*m in the study group and 283.6 ± 52.4 *μ*m in the control group, indicating that the SFCT is lower in the eyes of CHF patients compared to age- and gender-matched control subjects. A compensatory peripheral vasoconstriction develops in CHF in order to maintain satisfactory blood pressure and vasoconstriction in orbital and choroidal vessels causes a lower SFCT in CHF patients [30].

Physiological factors may have an effect on choroidal thickness [29]. Aging is one of the most important factors associated with changes in choroidal thickness. In our study, the mean SFCT of all eyes was 216.2 ± 46.8 *μ*m. Several studies have reported mean SFCTs between 261 ± 88 *μ*m and 354 ± 111 *μ*m in different age groups [31–33]. Chen et al. evaluated the interocular symmetry of macular choroidal thickness using enhanced depth imaging optical coherence tomography in 100 eyes of 50 healthy subjects [34]. They concluded that there was no significant difference in the intraindividual choroidal thickness.

In our study, there was a statistically significant positive correlation between the percentage of internal carotid artery stenosis and SFCT (*r* = 0.44, *P* = 0.03), but there was no significant correlation between SFCT and IOP and OPP, indicating that the subfoveal choroidal thickness increases as the percentage of stenosis increases. Recent studies have already indicated that the thickness of the choroid changes in the course of some disorders such as the glaucoma. Kubota et al. revealed decreased choroidal thickness in eyes with secondary angle-closure glaucoma [35]; Arora et al. reported that choroidal thickness was significantly greater in the angle-closure glaucoma group than in the open angle glaucoma group and normal subjects, with no significant difference between eyes with open angle glaucoma and normal subjects [36]. Hosseini and colleagues revealed that, except for the temporal region, choroidal thickness did not differ between glaucomatous and control eyes [37]. A recent study conducted by Wei et al. investigated the association between choroidal thickness and IOP and OPP values, and similar to our results, no statistically significant correlation was found in healthy subjects [38]. Altinkaynak et al. also evaluated the correlation between choroidal thickness and IOP and OPP values in congestive heart failure (CHF) patients, and they found no significant correlation between SFCT and IOP and OPP in CHF patients [30].

In conclusion, subfoveal choroidal thickness was found to be high in the presence of more than 70% ICA stenosis in the current study. Spectral-domain OCT is a useful, noninvasive, diagnostic tool for the assessment of the choroid and the evaluation of chorioretinal vascular changes in an aging population with various atherosclerotic risk factors. Further studies with larger cohorts and longer follow-up times are needed to evaluate the effects of various atherosclerotic risk factors on choroidal thickness.

## Figures and Tables

**Table 1:** The demographic features and baseline characteristics of patients.

Number of patients (*n*)	21
Age (mean ± SD)	71.9 ± 10.8 (52–84)
Sex, male/female	13/8
Hypertension (*n*, %)	21 (100%)
Hypercholesterolemia (*n*, %)	21 (100%)
Coronary artery disease (*n*, %)	16 (76.1%)
Tobacco use (*n*, %)	18 (85.7%)
Sleep apnea syndrome (*n*, %)	9 (42.8%)
History of stroke (*n*, %)	12 (57.1%)
SBP, mmHg [mean ± SD (range)]	104 ± 1.5 (90–120)
DBP, mmHg [mean ± SD (range)]	81.3 ± 1.3 (65–90)
MABP, mmHg [mean ± SD (range)]	86.3 ± 1.1 (70–95)

SBP: systolic blood pressure; DBP: diastolic blood pressure; MABP: mean arterial blood pressure; and SD: standard deviation.

**Table 2:** Subfoveal choroidal thickness and other clinical measurements in both groups.

	Group 1	Group 2	*P* value
SFCT, mean ± SD	231.9 ± 44.6	216.2 ± 46.8	*P* = 0.028
Percentage of ICA stenosis (%), mean ± SD	74 ± 4.9 (70–85)	47.5 ± 7.7 (35–60)	
PSV (cm/sec), mean ± SD	235.53 ± 7.8	118.95 ± 16.1	*P* < 0.01
Refractive error [mean ± SD (diopters)]	−0.27 ± 1.6	−0.34 ± 1.8	*P* = 0.530
IOP mmHg [mean ± SD, (range)]	15.2 ± 1.1 (12–18)	14.9 ± 18 (11–17)	*P* = 0.546
OPP mmHg [mean ± SD, (range)]	41.8 ± 1.6	40.9 ± 1.3	*P* = 0.435

ICA: internal carotid artery; SFCT: subfoveal choroidal thickness; PSV: peak systolic velocity; OPP: ocular perfusion pressure; IOP: intraocular pressure; and SD, standard deviation; *P* value of less than 0.05 was considered to be significant.

**Table 3:** Spearman rank correlation analyses between the subfoveal choroidal thickness and the percentage of ICA stenosis, IOP, and OPP.

	SFCT	*P* value
	Spearman rank correlation	
Percentage of ICA stenosis	*r* = 0.893	*P* = 0.001
IOP	*r* = 0.089	*P* = 0.693
OPP	*r* = 0.009	*P* = 0.726

SFCT: subfoveal choroidal thickness; ICA: internal carotid artery; OPP: ocular perfusion pressure; and IOP: intraocular pressure; *P* value of less than 0.05 was considered to be significant.
